# Update to living systematic review on prediction models for diagnosis and prognosis of covid-19

**DOI:** 10.1136/bmj.m2810

**Published:** 2020-07-22

This living systematic review by Wynants and colleagues (*BMJ* 2020;369:m1328) has been updated. For the latest update, visit doi:10.1136/bmj.m1328.

